# Effect of Landscape Pattern on Insect Species Density within Urban Green Spaces in Beijing, China

**DOI:** 10.1371/journal.pone.0119276

**Published:** 2015-03-20

**Authors:** Zhimin Su, Xiaoma Li, Weiqi Zhou, Zhiyun Ouyang

**Affiliations:** State Key Laboratory of Urban and Regional Ecology, Research Center for Eco-Environmental Sciences, Chinese Academy of Sciences, Beijing, China; Swedish University of Agricultural Sciences, SWEDEN

## Abstract

Urban green space is an important refuge of biodiversity in urban areas. Therefore, it is crucial to understand the relationship between the landscape pattern of green spaces and biodiversity to mitigate the negative effects of urbanization. In this study, we collected insects from 45 green patches in Beijing during July 2012 using suction sampling. The green patches were dominated by managed lawns, mixed with scattered trees and shrubs. We examined the effects of landscape pattern on insect species density using hierarchical partitioning analysis and partial least squares regression. The results of the hierarchical partitioning analysis indicated that five explanatory variables, i.e., patch area (with 19.9% independent effects), connectivity (13.9%), distance to nearest patch (13.8%), diversity for patch types (11.0%), and patch shape (8.3%), significantly contributed to insect species density. With the partial least squares regression model, we found species density was negatively related to patch area, shape, connectivity, diversity for patch types and proportion of impervious surface at the significance level of *p* < 0.05 and positively related to proportion of vegetated land. Regression tree analysis further showed that the highest species density was found in green patches with an area <500 m^2^. Our results indicated that improvement in habitat quality, such as patch area and connectivity that are typically thought to be important for conservation, did not actually increase species density. However, increasing compactness (low-edge) of patch shape and landscape composition did have the expected effect. Therefore, it is recommended that the composition of the surrounding landscape should be considered simultaneously with planned improvements in local habitat quality.

## Introduction

Rapid urbanization is generally considered to be one of the main drivers of biodiversity loss, resulting in major local extinctions, decreases in native species diversity, changes in species composition and outbreaks of individual species [1, 2]. When identifying environmental determinants, most ecologists agree that a reduction in habitat area [3–5], increased habitat isolation [6] and landscape heterogenization [7] are major factors in decreasing species richness. Some studies also suggest that land use type affects species diversity [8–10]. However, the relative importance of different landscape factors is the subject of debate. Given that these factors are often interdependent, it is necessary to disentangle the role of different environmental pressures on biodiversity to develop strategies to mitigate the potential detrimental impacts of urbanization.

China’s urbanization reached a historic point in 2011 when, for the first time, the urban population exceeded the rural population [11], a rapid trend that will be maintained in the coming 20 years. Beijing, as the capital and second largest city of China, is a typical example of urban development. The process of urbanization has progressed sharply in recent decades, with urban areas expanding nearly seven-fold over the last 30 years, more so than during the previous 3000 years [12, 13]. Therefore, understanding the biodiversity–urbanization relationships in Beijing may provide a suitable model for other cities in China, and even the world.

In the present study, we used a standardized sampling regime to estimate the species density of insect communities within urban green spaces in Beijing. All datasets were rarefied to the same number of samples, which allowed a valid comparison of species density. Species density refers to the number of species per unit area or average species number per sample (each sample covered the same spatial area) [14], which is usually referred to as ‘species richness’ in most studies [15]. Specifically, species density compares the number of species per unit area, while species richness compares the number of species per specific number of individuals. To a certain extent, species density represents the effectiveness of land-resource use with respect to biodiversity. However, land resources are scarce in urban areas and it is impractical to have large green spaces for species conservation within modern cities. Therefore, it is important to establish the efficacy of land use for insect biodiversity and the preservation of green spaces within urban areas. Species density seems more appropriate to be used to compare communities for conservation purposes and applied problems, rather than species richness which is commonly for testing models and evaluating theoretical predictions [16]. The following questions were addressed: (i) Does insect species density correlate with urban landscape pattern? (ii) Which type of landscape metrics contributed more to the variation in species density, landscape composition, landscape configuration, or local patch characteristics? (iii) How do the significant explanatory variables affect species density pattern?

## Materials and Methods

### Ethics statement

No specific permits were required for the described field study and our study did not involve endangered or protected species. Voucher specimens were deposited in the Research Center for Eco-Environmental Sciences, Chinese Academy of Sciences.

### Study sites

**Fig 1:**
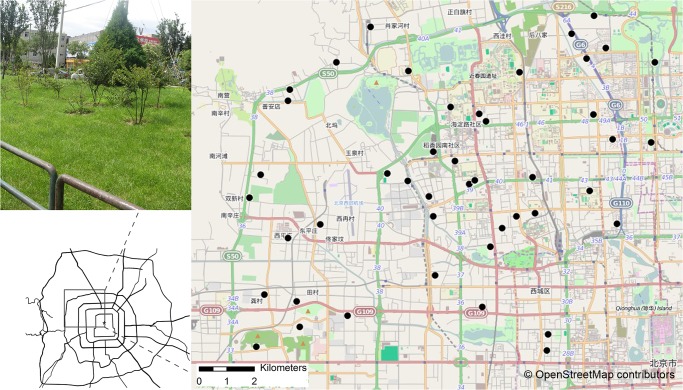
Map of study area within Beijing. The location of 45 green patches surveyed in this study shown as circular dots. The sample photograph in the upper left shows the vegetated and managed conditions of most surveyed green patches.

This study was carried out in the northwest quadrant of a built-up area of Beijing (Fig. 1). Beijing (39°28′–41°05′ N, 115°20′–117°30′ E) is the capital and second largest city of China (with a population of 20.69 million in 2012). It occupies an area of 16,410 km^2^ with 3377 km^2^ being designated as built-up areas [17]. Beijing has a typical concentric urban expansion pattern, forming a clear ring-shaped pattern from the city center outwards. Green space accounted for 31% of the land cover within the fifth ring road [18].

By random selection from a land use classification map using Hawth’s Analysis Tools for ArcGIS [19] and subsequent extensive field surveys in the study area, we identified 45 suitable green study patches along a gradient of landscape pattern (Fig. 1). The selected patches varied from 149 to 46582 m^2^ in size (Table 1). The vegetation was mainly composed of managed lawn mixed with some canopy species, woodland and shrubby understory. Most of the patches were intensively managed, including frequent weed removal and tree care (see photograph in the upper left of Fig. 1).

**Table 1:** Landscape metrics considered to be potential predictors for insect species density (variables in landscape composition and configuration were investigated within the 500 m radius around each surveyed patch).

Type	Metric (Abbr.)	Description* (Unit)	Range	Mean
Patch characteristic	Patch area (Area)	Patch area of the surveyed green patch (m^2^)	149.0–46582.0	5549.9
	Patch shape index (ShapeInd)	Shape index of the surveyed green patch	1.114–3.373	1.758
		$\text{ShapeInd}=\frac{p}{2\sqrt{\pi \cdot a}}$		
Landscape composition	Percentage of vegetated land (PVEG)	Proportion of the landscape occupied by vegetated land (%)	19.687–70.741	41.862
		$\text{PVEG}=\frac{\sum a_{\text{veg}}}{A}\times 100$		
	Percentage of impervious surface (PIS)	Proportion of the landscape occupied by impervious land (%)	26.187–80.205	56.105
		$\text{PIS}=\frac{\sum a_{\text{ips}}}{A}\times 100$		
	Shannon’s diversity index (SHDI)	Shannon’s diversity index of all patch types in the landscape	0.504–0.978	0.718
		SHDI=−∑(P⋅lnP); $P=\frac{a}{A}\times 100$		
Landscape configuration	Largest patch index (LPI)	Proportion of the landscape occupied by the largest green patch (%)	2.630–66.251	20.804
		$\text{LPI}=\frac{\text{max}\left(a_{\text{veg}}\right)}{A}\times 100$		
	Area-weighted mean of shape index (SHAPE_AM)	Area-weighted mean value of shape index of all green patches	2.059–8.357	4.652
		$\text{SHAPE\_AM}=\sum \left[\left(\frac{p_{\text{veg}}}{\text{min}\;p_{\text{veg}}}\right)\cdot \left(\frac{a_{\text{veg}}}{\sum a_{\text{veg}}}\right)\right]$		
	Mean of proximity index (PROX_MN)	Mean value of proximity index of all green patches	58.392–5323.390	952.451
		$\text{PROX\_MN}=\frac{1}{n}\times \sum \frac{a_{\text{veg}}}{h_{\text{veg}}^{2}}$		
	Area-weighted mean of proximity index (PROX_AM)	Area-weighted mean value of proximity index of all green patches.	48.477–3275.346	527.141
		$\text{PROX\_AM}=\sum \left[\left(\sum \frac{a_{\text{veg}}}{h_{\text{veg}}^{2}}\right)\cdot \left(\frac{a_{\text{veg}}}{\sum a_{\text{veg}}}\right)\right]$		
	Mean of Euclidean nearest neighbor distance (ENN_MN)	Mean distance to the nearest neighboring green patch based on the edge-to-edge distance (m)	10.462–20.266	14.275
		$\text{ENN\_MN}=\frac{1}{n}\times \sum h_{\text{veg}}$		
	Connectivity index (the vegetated area connected by ≤ 5 m of cleared land) (Conn_5m)	The number of functional joining between green patches, where each pair of patches is connected by ≤ 5 m of cleared land (%)	0–1.111	0.231
		$\text{Conn\_5m}=\left[\frac{\sum c_{\text{veg}}}{\frac{n\left(n-1\right)}{2}}\right]\times 100$		

* *a* is the patch area.

*A* is the total landscape area.

*c* is joining between two patches (0 = unjoined, 1 = joined) of the same patch type, based on a user-specified threshold distance (5 m in this study).

*h* is the distance between two patches, based on patch edge-to-edge distance, computed from cell center to cell center.

*n* is the number of patches.

*p* is the patch perimeter.

*P* is the proportion of the landscape occupied by one patch type.

veg indicates vegetated land.

ips indicates impervious surface.

### Insect sampling and species density acquisition

A total of 5–30 quadrats were sampled in each patch, depending on the patch area. We used an open-top and -bottom cage (length: 70 cm, width: 70 cm, height: 60 cm) to fix a sampling quadrat on the lawn and also to prevent insects from escaping. Quadrats were randomly distributed on the groundcover vegetation and at least 3 m away from each other within a green patch.

During July 2012, insects were sampled by suction trapping. Sampling was not performed during rainy periods or when heavy dew was present on the lawns. The suction device consisted of a vacuum cleaner (VK140-1, with a 60000-rpm motor and a WB14 nozzle of 60 cm^2^ in area; Vorwerk Elektrowerke GmbH) and a small electricity generator (2GF, rated power: 2000 W; Chongqing Zhoutai Power Machine Co., Ltd.). All insects on the plants within the quadrat (in the cage) were suctioned into a bag fixed behind the nozzle. One bag was reserved for each sampling quadrat. The specimens caught were preserved in 99% ethanol and identified to species or morphospecies level in the laboratory according to [20] and [21].

Species density, referred to the number of insect species per specific number of samples/quadrats (i.e., species number per unit area), was used as the response variable in this paper. To have the same sampling effort for each patch, data on insects were re-assessed by sample-based rarefaction using EstimateS 8.2 [22]. Comparisons will be in terms of species density when sample-based rarefaction curves are simply left scaled by accumulated sample number [16]. Therefore, here, Sobs (Mao Tau; i.e. the expected richness function in EstimateS 8.2) corresponding to five samples (i.e., the maximum sampling effort for the smallest patch) was taken as insect species density (i.e., species number per 2.45 m^2^) for each green patch.

### Environmental variables

A total of 11 environmental variables were calculated based on a land cover map using Fragstats 3.3. The map included four land cover types (Fig. 1): impervious land, vegetated land, water and bare land, and was classified from ALOS (*Advanced Land Observing Satellite*) images (taken in 2010 with 2.5 m resolution) using an objected-based classification method with Definiens Developer 7.0. The classification accuracy of the land cover map was 87% (Kappa value).

All environmental variables, sorted into three categories—local patch characteristics, landscape composition and landscape configuration—were considered in this study. Variables of the two latter categories, i.e., landscape composition and landscape configuration, were calculated within a radius of 500 m around each surveyed green patch. Table 1 lists detailed descriptions and equations for these variables [23].

1. Local patch characteristics describe the features of each surveyed green spaces, including patch area (Area) and patch shape index (ShapeInd). The latter considers the relationship of perimeter and area, which expands as the amount of edge increases. Biodiversity at the scale of habitat patch basically depended on local patch characteristics [24, 25].
2. Landscape composition variables encompassed all land-cover types within a 500-m radius of the specific green patches, including percentage of vegetated land (PVEG), percentage of impervious surface (PIS), and Shannon’s diversity index (SHDI). The amount and structure of different land cover types within the landscape may influence habitat quality and, thus, affect the survival/persistence of insect species [26, 27].
3. Landscape configuration variables specifically referred to patches of vegetated land, including largest patch index (LPI), area-weighted mean of shape index (SHAPE_AM), mean of proximity index (PROX_MN), area-weighted mean of proximity index (PROX_AM), mean of Euclidean nearest neighbor distance (ENN_MN), and connectivity index (the vegetated area connected by ≤ 5 m of cleared land) (Conn_5m). The proximity index quantifies the spatial context of the focal patch in relation to its neighbors of the same class [28]. Landscape configuration can affect a variety of processes such as species dispersal and source-sink dynamics [27].

### Statistical analyses

Prior to analyses, both response variable (i.e., species density) and explanatory variables were checked for normality using the Kolmogorov–Smirnov test and then normalized if necessary. Data on four of the landscape variables (i.e., area, LPI, PROX_MN and PROX_AM) were log-transformed and the connectivity variable was square root-transformed. All the analyses below were conducted in R Version 3.1.1 [29].

Initially, spatial autocorrelation in insect species density within the urban green patches was checked using Moran’s *I* test. Then, hierarchical partitioning (HP) was used to identify and distinguish explanatory variables, whose independent correlation with insect species density may be important, from variables that have little independent effect. HP is a useful method in which all possible combinations of variables are assessed to determine the independent contribution of each variable to model fit. We included all the 11 environmental metrics as explanatory variables and used *R* ^2^ as the goodness-of-fit measure. Randomization was used to assess the significance of the independent contributions to variance (based on 100 permutations). Explanatory variables obtaining *Z*-scores ([observed – mean randomization]/SD randomization) higher than 1.65 (upper 95% confidence limit) were considered a significant influence on the response variable [30]. This analysis was carried out using the package ‘hier.part’.

Then, partial least squares regression (PLSR) was used to investigate the relationship between species density and all the 11 explanatory variables some of which were highly correlated (S1 Table). PLSR is particularly useful in analyzing ecological data with strongly collinear independent variables [31] and explains the maximum covariation between species density and explanatory variables. Before performing PLSR, we standardized all the variables to a mean of zero and variance of one. The plsr procedure was firstly run to fit a model with 11 components, including leave-one-out (LOO) cross-validated predictions [32]. The optimal component number of final PLSR model was determined by the minimum value of root mean squared error of prediction (RMSEP). The jack-knife *t*-test was used to examine the statistical significance of coefficient for each explanatory variable in the model [33]. This analysis was carried out using the package ‘pls’.

Finally, regression tree analysis [34] was used to further identify the influential explanatory variables and describe the distribution pattern of insect species density. The trees were constructed by repeatedly splitting the response variable (i.e., insect species density) using binary recursive partitioning, including all the 11 environmental metrics as explanatory variables. To obtain the best tree, trees were pruned using 10-fold cross-validation with the one-SE (standard error) rule, so that the final tree was the smallest within one SE of the minimum model. Total variance, explained by the best single tree, was calculated as *R* ^2^ = 1—relative error. This analysis was completed using the package ‘rpart’.

## Results

In the 45 urban green patches, we collected 116 species / morphospecies from 10 orders, 58 families with 3561 specimens (Table 2). Species density varied from four to 17 species per 2.45 m^2^ (area of five samples), with an average value of over 8.0 across 45 green patches. Hemiptera and Diptera were the two most common and abundant groups, which were found in all study patches, and both accounted for over 1/3 of total insect abundance. The third most abundant order was Hymenoptera, accounting for 15% of total abundance, which was dominated by parasitoid wasps.

**Table 2:** List of the 10 insect orders recorded from 45 urban green patches.

Order	No. families	No. species / morphospecies	No. individuals
Hemiptera	12	36	1273
Diptera	13	15	1260
Hymenoptera	10	17	542
Orthoptera	9	14	367
Coleoptera	9	25	77
Lepidoptera	1	3	20
Mantodea	1	2	15
Odonata	1	1	4
Neuroptera	1	2	2
Dermaptera	1	1	1
Total	58	116	3561

Moran’s *I* tests indicated that there was no significant spatial autocorrelation across surveyed patches for insect species density (Moran’s *I* = −0.015, *P* = 0.465).

**Fig 2:**
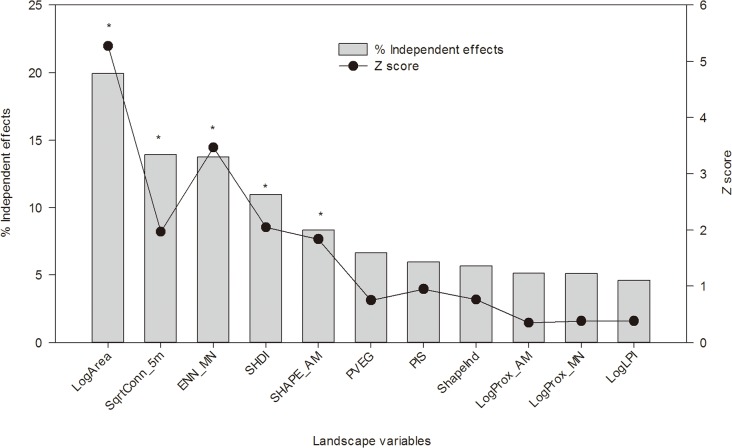
The independent contribution of each landscape metric to model fit for insect species density. The hierarchical partitioning model includes all variables indicated in the figure. Statistically significant variables at *Z*-score ≥ 1.65 are indicated by an *asterisk*. The abbreviations of landscape metrics are as shown in Table 1. In addition, LogArea, LogProx_AM, LogProx_MN and LogLPI are logarithms to the base 10 of Area, Prox_AM, Prox_MN and LPI, respectively; SqrtConn_5m is square root of Conn_5m.

HP for species density revealed that LogArea (with 19.9% independent effects), SqrtConn_5m (13.9%), ENN_MN (13.8%), SHDI (11.0%) and SHAPE_AM (8.3%) made a significant independent contribution to the variance, as explained by the full model (*Z*-scores ≥ 1.65) (Fig. 2). Patch area made the greatest independent contribution to model fit.

**Fig 3:**
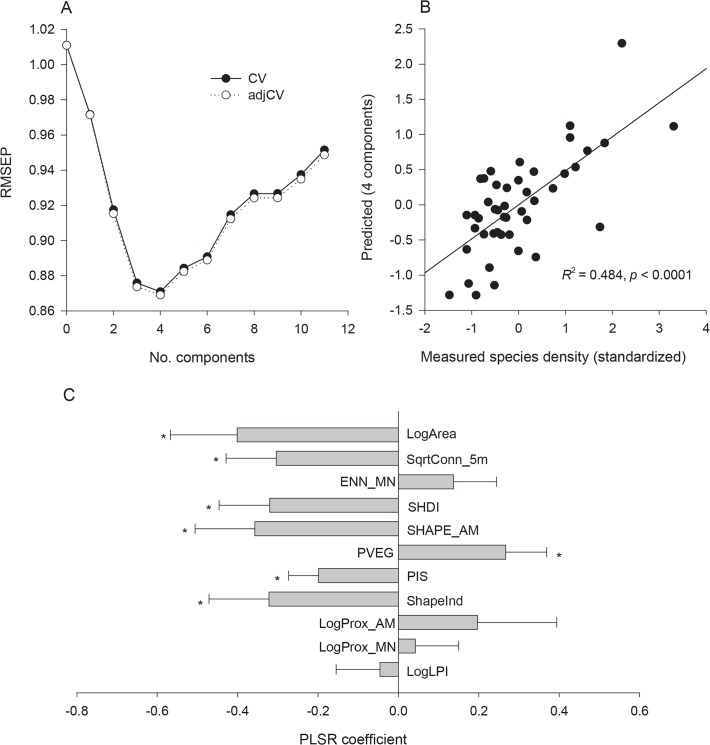
Partial least squares regression (PLSR) for insect species density with the 11 landscape metrics. (A) Cross-validated root mean squared error of prediction (RMSEP) curves. (B) Measured species density (standardized) versus values predicted by the PLSR model with four latent components. (C) Regression coefficients (with standard errors) for the PLSR model with four latent components. An *asterisk* indicates significant variables at *p* ≤ 0.05 estimated using jack-knife *t*-test. The abbreviations of landscape metrics are as shown in Table 1. In addition, LogArea, LogProx_AM, LogProx_MN and LogLPI are logarithms to the base 10 of Area, Prox_AM, Prox_MN and LPI, respectively; SqrtConn_5m is square root of Conn_5m.

The PLSR model with four components had the minimum RMSEP value (RMSEP_adjCV_ = 0.869) (Fig. 3A), and it can predict 48.4% of the variation in the insect species density (*p* ≤ 0.0001) (Fig. 3B). Seven of the explanatory variables, i.e. LogArea, SqrtConn_5m, SHDI, SHAPE_AM, PVEG, PIS and ShapeInd, were all significant (*p* ≤ 0.05) in explaining the variation of species density (Fig. 3C). Among all the significant variables, six ones (i.e. LogArea, SqrtConn_5m, SHDI, SHAPE_AM, PIS and ShapeInd) had negative effects on insect species density, with coefficients of −0.401, −0.304, −0.320, −0.357, −0.199 and −0.322, respectively; only PVEG was positively related to species density, with a coefficient of 0.267.

**Fig 4:**
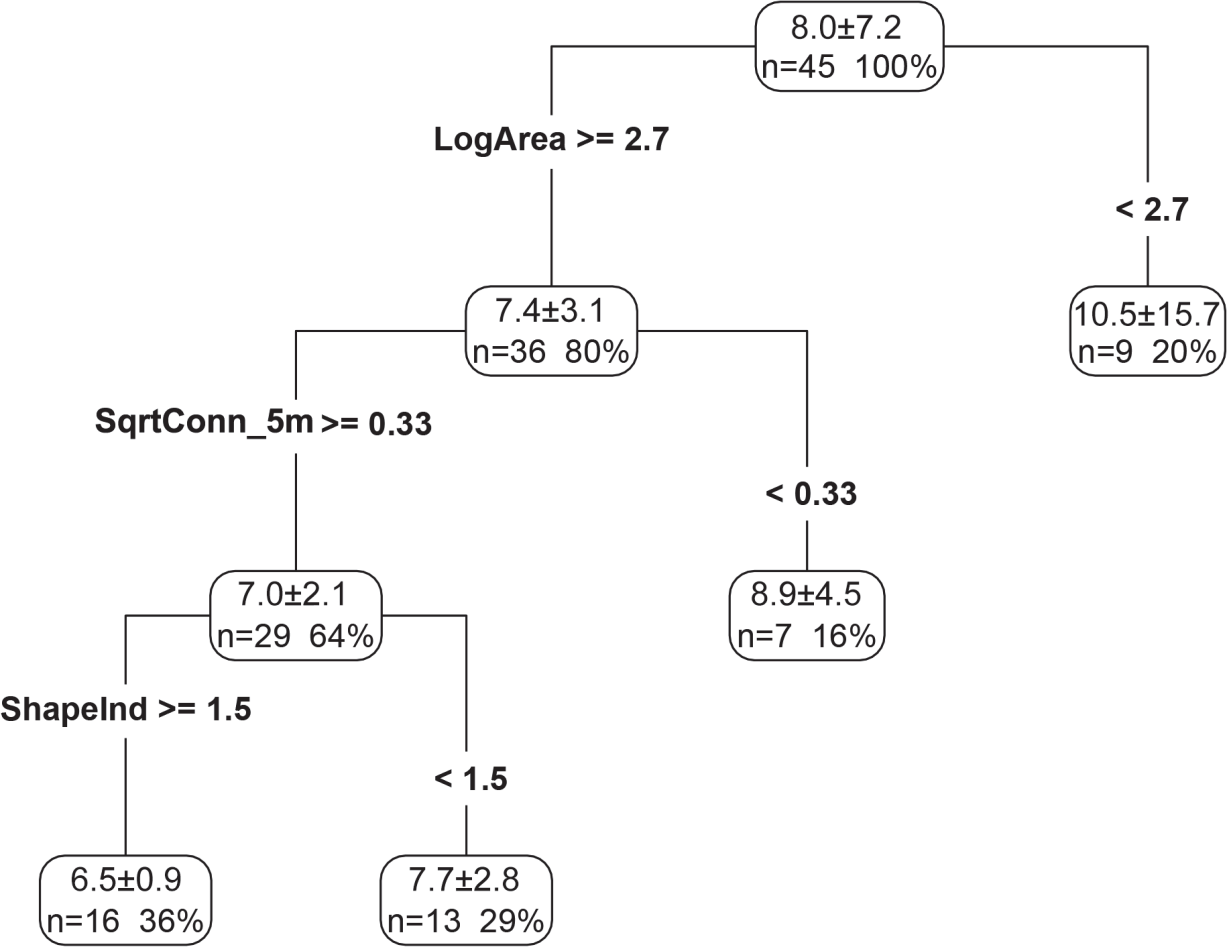
Regression tree analysis of insect species density in each green patch. Each node of the tree is described by the splitting variable and its split value (LogArea, SqrtConn_5m, ShapeInd, mean and SE of species density, the number and percentage of patches at that node). The total variance explained is *R* ^2^ = 0.313. The abbreviations of landscape metrics are as shown in Table 1. LogArea is logarithm to the base 10 of Area; SqrtConn_5m is square root of Conn_5m.

Regression trees lent further support to the fact that LogArea, SqrtConn_5m and ShapeInd had an overwhelming effect on insect species density (Fig. 4). The best tree explained 31.3% of variance, of which 21.8% was attributed to LogArea, 6.4% to SqrtConn_5m, and 3.1% to ShapeInd. A lower species density was supported by a larger patch area, connectivity and shape index, reaching lowest values in green patches with areas >501.2 m^2^ (Log_10_ (Area) ≥ 2.7), a connectivity index > 0.11% (Sqrt (Conn_5m) ≥ 0.33) and a shape index exceeding 1.5. The highest species density was found in green patches with areas < 500 m^2^, regardless of the connectivity or shape index.

## Discussion

It was found that insect species density was significantly affected by patch characteristics (patch shape and area of urban green space), landscape composition (Shannon diversity index) and landscape configuration (Connectivity).

### Patch characteristics

In the present study, all the three statistical analysis methods (HP, PLSR and regression tree) highlighted the fact that patch area had a predominant effect on insect species density within urban green patches. However, it was an unexpected result that insect species density was negatively correlated with patch area in the PLSR model, i.e., species density decreased as patch area increased. The regression tree provided further positive evidence for this result. The results were not biased by the effects from variation in land use type, established via analysis of covariance (ANCOVA) (S2 Table).

In this study, species density was positively affected by individual density (S1 Fig.). Moreover, individuals were less dense (i.e., fewer individuals per unit area) in larger patches, although it was not at statistically significant level of *p* < 0.05 for the regression model between individual density and patch area (S2 Fig.). Accordingly, we may make an assumption that insect individuals disperse more widely in larger urban green patches (we did not observe any obviously aggregative distribution for the caught taxa in the field survey), causing a decrease in individual number per unit area. Some previous studies also found fewer individuals per unit area in larger areas [35]. As a result, species density decreased as patch area increased. This result suggests that large green patches may be ineffective in species conservation in the highly managed lawns within modern cities.

According to the general principles of shape and function, a compact shape (with a lower value of patch shape index in this study) should have a smaller proportion of edge within a unit area available for the maintenance of species, while shapes with a greater edge should tend to promote exchange between the inner patch and outer environment [36]. However, there is a lack of empirical evidence to support this. In this study, habitat patch shape, quantified by a shape index at a patch level and an area-weighted mean of shape index at a class level, had important negative effects on insect species density. This implies that a more irregular shape would intensify the edge effect of green patches, aggravate the disturbance of species within the habitat and, thereby, reduce species density.

### Landscape composition

Biodiversity depends not only on the properties of a single ecosystem, but also on spatial interaction between multiple ecosystems and on anthropogenic elements such as roads, buildings and other artifacts [37]. The composition of the landscape matrix is commonly considered as habitat quality, which plays an important role in the persistence of biodiversity within urban areas [5, 8, 38]. We found that the diversity of landscape (SHDI) negatively affected insect species density. It is understandable that increasing the number of different patch types may intensify habitat fragmentation. A number of previous studies have also suggested that the surrounding landscape obviously influences the area of suitable habitats and presumably affects the ability of insects to move and disperse [39]. In the PLSR model, insect species density increased with increased PVEG, but decreased with increased PIS. Decreased matrix permeability may be problematic for the survival of insect species partly because most insect species are more or less associated with soil environments. For example, some insect species mainly inhabit soil during their life history (i.e., geobionts), some inhabit soil except for their adult stages (i.e., geophilous), while some hide, overwinter or pupate in soil [40].

### Landscape configuration

Habitat isolation is always taken into account in species conservation [6]. The degree of isolation can be quantified using the proximity index, connectivity index and nearest neighbor distance [41, 42]. According to our results, insect species density appeared to decrease with increasing connectivity with ≤ 5 m of cleared land. Similar to our findings, Rösch *et al*. (2013) recorded a reduction in species richness of leafhoppers in a simple landscape as connectivity increased [42]. It is difficult to explain why increasing connectivity failed to result in an increase in species density per site. It may in fact reflect the low connectivity of the green patches observed in this study, which varied from 0 to 1.11% (see Table 1). When patch isolation disrupts a predator’s ability to detect prey [43, 44], it may have a positive effect on the population density of prey or host species that are controlled by predators [25]. Control by natural enemies is often thought to enhance species coexistence and diversity [45], but not vice versa. On the other hand, increasing connectivity may strengthen emigration from local patches. As a result, species density may increase in isolated patches. The mechanism of tradeoff between predation and emigration rates should be studied in the future work.

## Conclusion

Urban green spaces can provide an important refuge for wildlife in urban areas. We studied the relationship between landscape pattern of green patches and biodiversity from a perspective of species density. Our results identified patch shape and area, landscape composition and connectivity as the dominant predictors of insect species density in urban green spaces. Furthermore, the results showed that improving some aspects of habitat quality typically thought to be important for conservation—such as patch area and connectivity—did not actually increase species density in this study. However, improving other aspects of habitat quality—such as compact (low-edge) patch shape and landscape composition—did have the expected effect. Thus, it is recommended that the composition of the surrounding landscape be considered simultaneously with planned improvements in local habitat quality. Further study will involve an investigation of the urbanization tolerance of different insect groups to achieve a deeper understanding of species’ responses to the landscape.

## Supporting Information

S1 Fig Relationship between species density and individual density. (TIF)

S2 Fig Relationship between individual density and patch area. (TIF)

S1 Table Pearson correlations among 11 environmental variables. (PDF)

S2 Table Analysis of covariance (ANCOVA) with species density (rarefied data to five samples) as dependent variable, land use type as explanatory factor and LogArea as covariate. (PDF)
