# Supplementary figures and images for: Effect of Landscape Pattern on Insect Species Density within Urban Green Spaces in Beijing, China
Source: PLoS One. 2015 Mar 20;10(3):e0119276. doi: 10.1371/journal.pone.0119276 (PMC4368726; doi:10.1371/journal.pone.0119276)

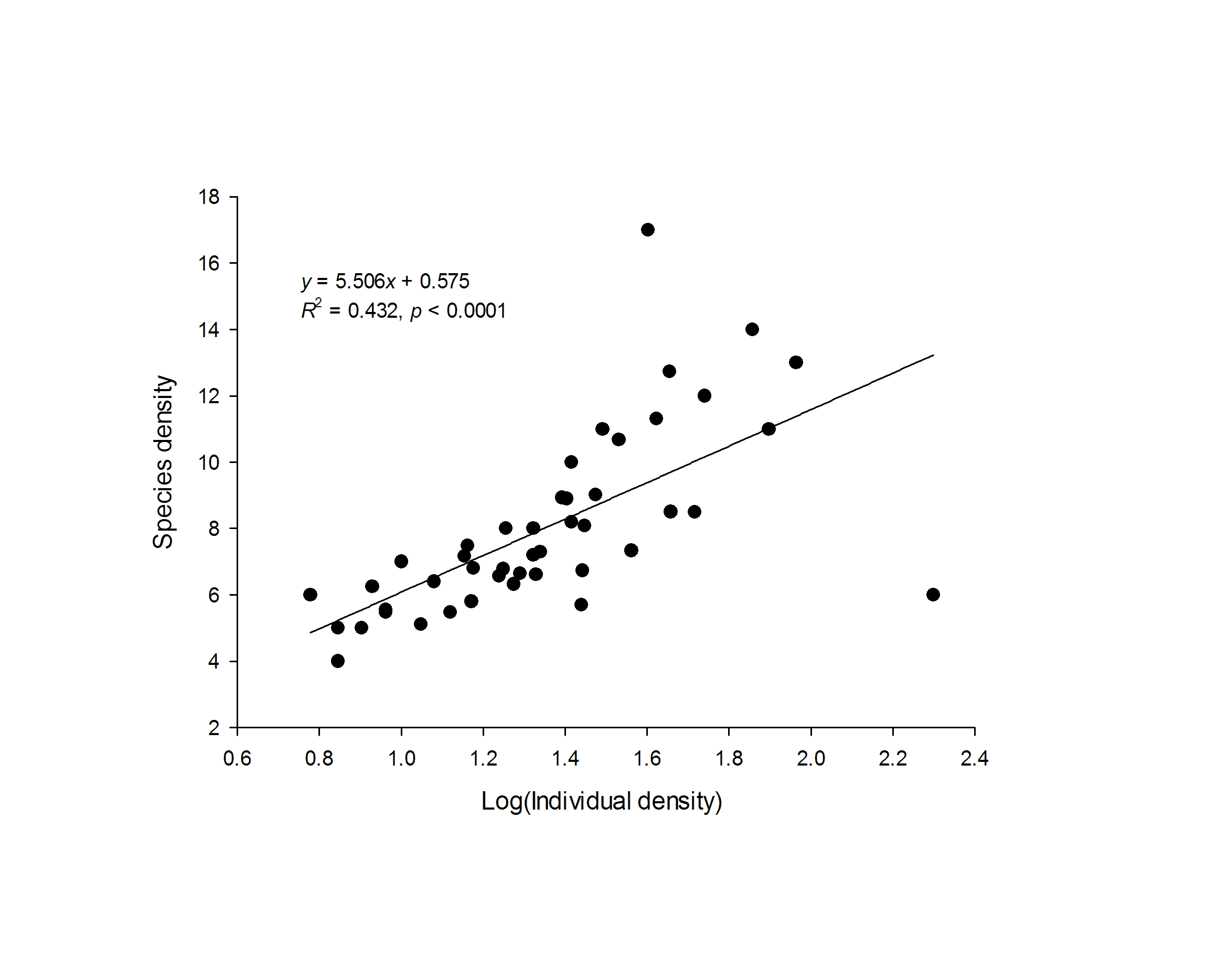

Supplement: S1 Fig — (TIF) [file pone.0119276.s001.TIF]

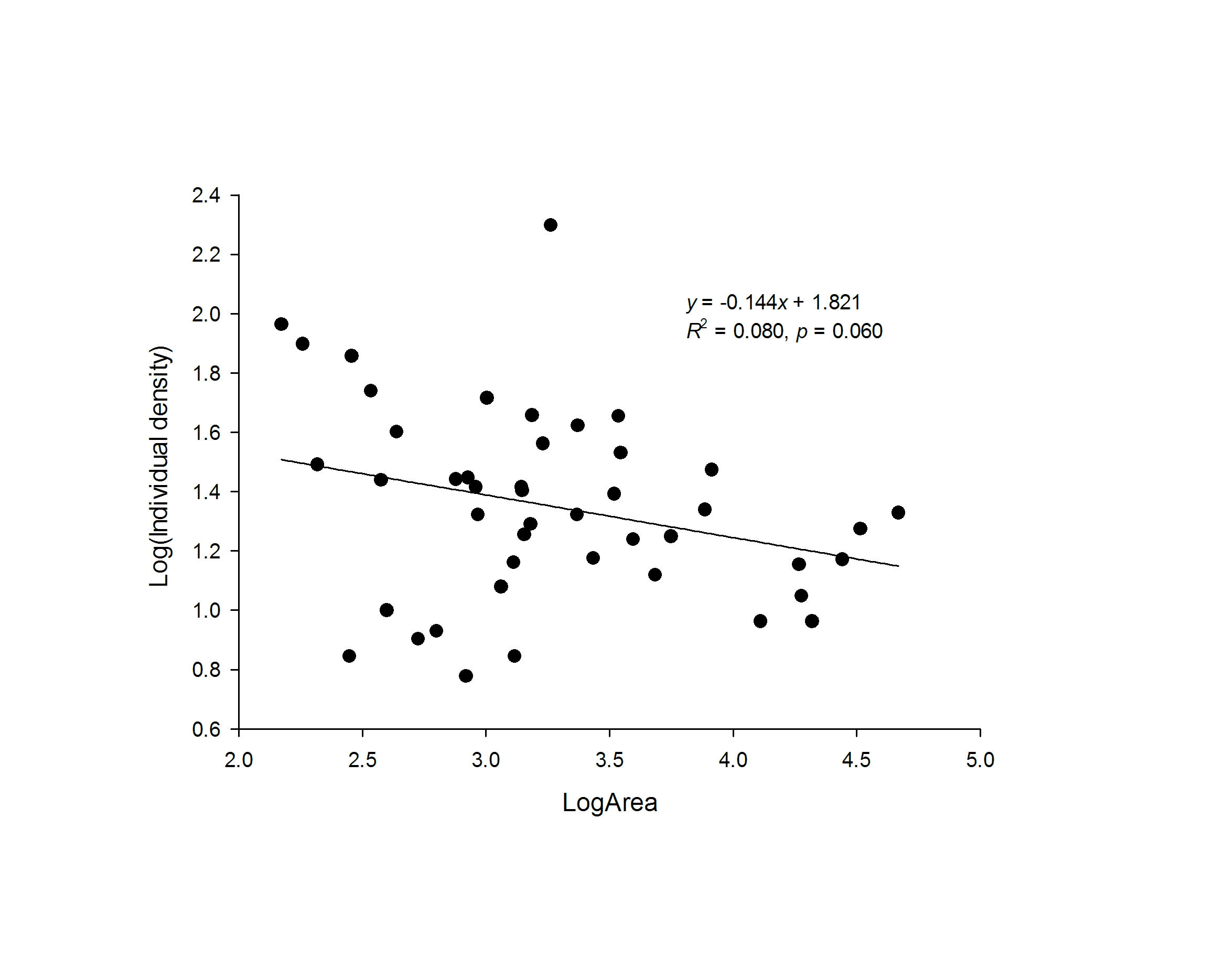

Supplement: S2 Fig — (TIF) [file pone.0119276.s002.TIF]
