# Supplementary material for: Effect of Landscape Pattern on Insect Species Density within Urban Green Spaces in Beijing, China
Source: PLoS One. 2015 Mar 20;10(3):e0119276. doi: 10.1371/journal.pone.0119276 (PMC4368726; doi:10.1371/journal.pone.0119276)
Supplement: S1 Table — (PDF) [file pone.0119276.s003.pdf]

Table S1. Pearson correlations among 11 environmental variables.

		LogArea	ShapeInd	PVEG	PIS	LogLPI	SHAPE_AM	LogProx_MN	LogProx_AM	ENN_MN	SHDI
ShapeInd	Pearson Correlation	-0.192									
	Sig. (2-tailed)	0.206									
PVEG	Pearson Correlation	0.360	-0.026								
	Sig. (2-tailed)	**0.015**	0.866								
PIS	Pearson Correlation	-0.395	0.051	-0.982							
	Sig. (2-tailed)	**0.007**	0.737	**<0.001**							
LogLPI	Pearson Correlation	0.218	-0.081	0.839	-0.821						
	Sig. (2-tailed)	0.149	0.597	**<0.001**	**<0.001**						
SHAPE_AM	Pearson Correlation	0.069	-0.217	0.332	-0.289	0.570					
	Sig. (2-tailed)	0.654	0.152	**0.026**	0.054	**<0.001**					
LogProx_MN	Pearson Correlation	0.318	-0.101	0.884	-0.857	0.914	0.646				
	Sig. (2-tailed)	**0.033**	0.511	**<0.001**	**<0.001**	**<0.001**	**<0.001**				
LogProx_AM	Pearson Correlation	0.237	-0.101	0.375	-0.365	0.266	0.535	0.490			
	Sig. (2-tailed)	0.117	0.507	**0.011**	**0.014**	0.077	**<0.001**	**0.001**			
ENN_MN	Pearson Correlation	-0.148	-0.062	-0.242	0.236	-0.132	-0.397	-0.253	-0.509		
	Sig. (2-tailed)	0.332	0.688	0.109	0.118	0.388	**0.007**	0.094	**<0.001**		
SHDI	Pearson Correlation	0.314	-0.132	0.320	-0.478	0.282	0.080	0.304	0.242	-0.197	
	Sig. (2-tailed)	**0.036**	0.387	**0.032**	**0.001**	0.060	0.603	**0.042**	0.109	0.194	
SqrtConn_5m	Pearson Correlation	0.457	-0.214	0.567	-0.585	0.434	0.294	0.543	0.329	-0.467	0.296
	Sig. (2-tailed)	**0.002**	0.159	**<0.001**	**<0.001**	**0.003**	**0.050**	**<0.001**	**0.027**	**0.001**	**0.048**

Value in bold indicates the significant correlation that $p \leq 0.05$.
