# Supplementary material for: Effect of Landscape Pattern on Insect Species Density within Urban Green Spaces in Beijing, China
Source: PLoS One. 2015 Mar 20;10(3):e0119276. doi: 10.1371/journal.pone.0119276 (PMC4368726; doi:10.1371/journal.pone.0119276)
Supplement: S2 Table — (PDF) [file pone.0119276.s004.pdf]

Effect of land use type

All study patches were classified into six types, including park, protection, institutional, residential, street green space and vacant land space, according to the standard for classification of urban green space (CJJ/T 85-2002) released by Ministry of Housing and Urban–Rural Development of China [1]. More details about the classification system can be found in Zhao et al. 2010 [2].

To examine the effect of land use type (park, protection, institutional, residential, street and vacant land) on insect species density, we carried out an ANCOVA with type of site as the explanatory factor and LogArea as the covariate using SPSS Version 18.0.

ANCOVA showed that land use type had no effect on species density when the effect of patch area was taken into account (F=1.386, p=0.251)

Table S2. Analysis of covariance (ANCOVA) with species density (rarefied data to five samples) as dependent variable, land use type as explanatory factor and LogArea as covariate.

Source	F	p	R^2
Corrected Model	2.544	0.036*	0.287
Intercept	24.454	<0.001***	
LogArea	5.302	0.027*	
Land use type	1.386	0.251	

* $p \le 0.05$, ** $p \le 0.01$, *** $p \le 0.001$

References

1. Ministry of Housing and Urban-Rural Development of China. Standard for Classification of Urban Green Space. Beijing: China Building Industry Press; 2002.
2. Zhao J, Ouyang Z, Zheng H, Zhou W, Wang X, Xu W, et al. Plant species composition in green spaces within the built-up areas of Beijing, China. Plant Ecol. 2010;209: 189–204.
